# Eco-evolutionary Red Queen dynamics regulate biodiversity in a metabolite-driven microbial system

**DOI:** 10.1038/s41598-017-17774-4

**Published:** 2017-12-15

**Authors:** Juan A. Bonachela, Meike T. Wortel, Nils Chr. Stenseth

**Affiliations:** 1 Marine Population Modeling Group, Department of Mathematics and Statistics, University of Strathclyde, Glasgow, G1 1XH Scotland UK; 2 Centre for Ecological and Evolutionary Synthesis (CEES), Department of Biosciences, University of Oslo, PO Box 1066 Blindern, Oslo, 0316 Norway; 3 Present Address: Department of Ecology, Evolution, and Natural Resources, 14 College Farm Rd, New Brunswick, NJ 08901, USA

## Abstract

The Red Queen Hypothesis proposes that perpetual co-evolution among organisms can result from purely biotic drivers. After more than four decades, there is no satisfactory understanding as to which mechanisms trigger Red Queen dynamics or their implications for ecosystem features such as biodiversity. One reason for such a knowledge gap is that typical models are complicated theories where limit cycles represent an idealized Red Queen, and therefore cannot be used to devise experimental setups. Here, we bridge this gap by introducing a simple model for microbial systems able to show Red Queen dynamics. We explore diverse biotic sources that can drive the emergence of the Red Queen and that have the potential to be found in nature or to be replicated in the laboratory. Our model enables an analytical understanding of how Red Queen dynamics emerge in our setup, and the translation of model terms and phenomenology into general underlying mechanisms. We observe, for example, that in our system the Red Queen offers opportunities for the increase of biodiversity by facilitating challenging conditions for intraspecific dominance, whereas stasis tends to homogenize the system. Our results can be used to design and engineer experimental microbial systems showing Red Queen dynamics.

## Introduction

In its original formulation, the Red Queen Hypothesis proposes that co-evolution among co-existing species can be perpetual, with no need for abiotic factors to sustain it^1^. Stripping the Red Queen (RQ) from some of its original controversial constraints (e.g. “zero sum rule”^1–3^) leads to a generic scenario in which evolutionary changes in one species impose selection pressures on others that necessarily adapt in order to avoid extinction; this response in turn influences the first species, thus generating dynamic fitness landscapes perpetually altered by the reciprocal evolutionary responses of interacting species^4^.

This less restrictive reading of the RQ Hypothesis has opened the door to interpreting a wider spectrum of situations as RQ dynamics. For example, although the original formulation intended to describe a macroevolutionary motif, the RQ is used to describe both macroevolutionary and microevolutionary patterns^2,3,5^. On the other hand, although the requirement for perpetual evolutionary “running” was initially interpreted as unbounded evolution (e.g. “arms race”), the RQ is currently also identified with co-evolutionary oscillations. In these oscillations, the evolutionary gradient experienced by one species changes sign due to the other co-evolving organisms, thus triggering a shift in the directional change of the focal adaptive traits^6^.

From a theoretical point of view, some degree of ecological asymmetry is required for models to show evolutionary oscillations^7^. Theories typically seek to find the conditions for evolutionary limit cycles (labeled as RQ dynamics) as opposed to evolutionarily stable strategies (ESS), which are identified with stasis^8^. However, in these models it is often difficult to realize when exactly these RQ oscillations are driven by evolutionary responses of the species involved, as the RQ really requires, or are instead caused by ecological components. The reason is that, in most cases, oscillations in the adaptive trait are linked to population-density oscillations^9,10^. In such cases, the co-evolving species alternate dominance unceasingly, i.e. one species rises and forces a simultaneous decline of the rest of the species’ densities; the RQ is identified as periodic changes in the relative frequency of the species involved, facilitating a winner-less scenario in which all species coexist. From this perspective, the RQ oscillations contribute to maintaining biodiversity^10^. Similarly, other models pinpoint the RQ as the cyclic alternation between a discrete number of strategies for finite populations using game-theory frameworks^11,12^, or as oscillations in the relative frequencies of alleles, with no need to keep track of population dynamics^13^. Moreover, models that show evolutionary oscillations in the adaptive traits’ value often impose very specific multi-parameter functional forms linking the species interaction strengths with the also-assumed dynamics for the adaptive trait (e.g. seminal^8,14^, or more complicated examples^9^). This specificity and required knowledge about the links between evolutionary and ecological dynamics contribute to a disconnect that prevents models from helping in the conception of experimental setups able to show RQ dynamics; for instance, because it may be difficult to match real traits or environmental conditions with model parameters and functions, or to find systems that fulfill such functional-response constraints. An experimental setup informed by and devised using models enables the understanding of fundamental aspects of the emerging experimental RQ dynamics such as the (ecological or evolutionary) mechanisms underlying such oscillations.

Here, we aim to fill this gap by studying a simple, generic model for microbial evolution able to show eco-evolutionary RQ oscillations. Based purely on biotic interactions, our model keeps track of each phenotype’s population dynamics but, differently from the traditional approach, RQ dynamics are not linked to nor do they result from population-density oscillations. In our model, intraspecific competition for resources drives interspecific ecological interactions among generic bacterial strains which, in turn, influence the evolutionary target imposed by intraspecific competition. This feedback loop triggers a perpetual change of the fitness landscape that gives rise to eco-evolutionary RQ dynamics. For the sake of concreteness, we focused on a specific theoretical scenario in which genetic engineering and metabolic byproducts were used to achieve the necessary chain of interactions, but the mechanisms presented here are general. We employed a generic functional dependence between intra- and interspecific interactions that allowed us to explore different drivers for the RQ in such a scenario. We explored how these different drivers alter the emergent RQ dynamics, and studied the role of the RQ in biodiversity regulation. Our model allows for a theoretical understanding of key aspects of the RQ, providing information about the triggering mechanisms and systems susceptible to show evolutionary oscillations in the lab and the real world.

## Methods

### Microbial model

We assume a system composed of several strains of a generic microbial unicellular organism and allow for intraspecific variability, i.e. multiple phenotypes per strain. Phenotypes within a strain interact via intraspecific resource competition, one of the most basic ecological interactions^15^. In addition, strains are connected by an interspecific interaction to be specified. Because interspecific interactions may contribute to destabilizing an interconnected system and lead to its collapse^16^, we set up a self-regulating interaction chain (an interspecific non-transitive cycle) to facilitate the survival of the strains.

### Intraspecific dynamics

We assume continuous cultures (i.e. chemostat conditions) and, therefore, the dynamics of the number of cells for any phenotype *i*, *N*_*i*_, are given by:

1 $$\frac{d{N}_{i}}{dt}=({\mu }_{i}-w){N}_{i},$$

where *μ* represents growth rate, and *w* is the dilution rate of the chemostat (only source of mortality, assuming that cell death rates are negligible compared to dilution). See Table S1 for list of symbols and units. Strains in the system are differentiated only by the resource they grow on, which prevents interspecific competitive exclusion. Experimentally, this can be achieved by using knock-out techniques for the genes that control the assimilation of the discarded resources. Thus, only the *S* phenotypes that belong to each individual strain compete for the same single resource. The dynamics for the availability of the strain-*a*-specific resource *A*, [*A*], are given by:

2 $$\frac{d[A]}{dt}=w({[A]}_{input}-[A])-\sum _{i\mathrm{=1}}^{{S}_{a}}{N}_{i}{\mu }_{i}/{Y}_{a},$$

(and similarly for strains *b*, *c*… and their respective resources, *B*, *C*, etc.). The first term represents the inflow and outflow of nutrients, and the second term represents the uptake of nutrient *A* by all the *S*_*a*_ phenotypes (*N*_*i*_ cells per phenotype *i*). The nutrient intake is assimilated as growth with a constant yield factor *Y*_*a*_ and, therefore, we can refer indistinctly to uptake or growth. We assume that the latter is given by the simple Monod formulation^17^; if phenotype *i* belongs to strain *a*:

3 $${\mu }_{i}=R(y)\frac{{\mu }_{ma{x}_{i}}[A]}{{K}_{i}+[A]},$$

where ${\mu }_{ma{x}_{i}}$ represents the associated maximum growth rate, *K*_*i*_ the half-saturation constant, and *R*(*y*) the interspecific interaction term.

### Interspecific interactions

We encode the effect that any possible interspecific interaction has on growth using the effective function, *R*(*y*), which depends on a driving biotic factor, *y*. Although not strictly necessary (see Supplementary Information, section S. VIII), we assume that this function is bounded and positively correlated with the biotic driver. For example, to describe the effect of strain *c* on phenotypes from strain *a* (Fig. S1):

4 $${R}_{a}({y}_{c})=\frac{{R}_{top}}{1+{e}^{-{k}_{R}({y}_{c}-{y}_{ref})}}+{R}_{min}\mathrm{.}$$

*R*_*min*_ defines the minimum value for *R*, and the maximum value is given by *R*_*max*_ = *R*_*top*_ + *R*_*min*_. The sensitivity of the interaction to the driver is provided by *k*_*R*_ and *y*_*ref*_, which control slope and location of the *R* curve respectively, and depend on the specifics of the biotic factor *y*. As indicated in S. IV, these are the only free parameters of the model.

### Non-transitive cycle

To set up the non-transitive cycle, an odd number of strains is required, and interspecific interactions need to be pairwise, directed, and negative: assuming 3 strains, *a* inhibits *b*; lower *b* growth benefits *c*; higher *c* growth in turn inhibits *a*, and so on. In such a chain, the directional change in *a* that initiates the cycle is reverted after one iteration (i.e. when *c* affects *a*), thus regulating and constraining interspecific interactions (see Fig. S2). Due to the positive role of *R* in cell growth (Eq. (3)), inhibition here means a decrease in *R*.

### Specific example and drivers

For the sake of concreteness, we focus on the bacterium *Escherichia coli* hereon. We assume that each strain synthesizes and excretes two metabolic byproducts that, in combination, inhibit totally or partially the growth of one and only one of the other strains: strain *c*’s inhibitor ratio only affects strain *a* phenotypes, *a* excretes inhibitors that can only be taken up by *b*, and *c* is the only strain affected by *b*-excreted inhibitors, achieving in this way the non-transitive cycle described in Fig. S2. Compound effects of metabolites have been reported such as the wild- *E. coli* growth-inhibition effect of valine requiring also isoleucine in a ratio 0.03:1^18,19^, and ratiometric sensors have been engineered in the past for *E. coli* ^20^. Inspired by, e.g. the positive correlation between *E. coli* cell growth and excretion of the inhibitor acetate^21^, here good growth performance from a phenotype increases its metabolite ratio and therefore increases inhibition for the target strain. Thus, we assume that the biotic factor driving the interaction depends on growth efficiency, negatively correlated with the growth half-saturation constant (see Eq. (3)). For example:

5 $${y}_{c}=\frac{{K}_{c}}{{K}_{re{f}_{c}}}\mathrm{.}$$

Higher growth efficiency from *c* (i.e. lower *K*_*c*_) reduces *R*_*a*_(*y*_*c*_) and, therefore, *a*’s growth. With no loss of generality, we use as representative *K* for the strain that of the dominant phenotype, typically overpowering the population average. To facilitate comparison across drivers, we use a normalization factor provided by the trade-off existing between the half-saturation constant and maximum growth rate (see below). Another plausible driver considers the relative growth efficiency (e.g. the inhibitory effect of the metabolite ratio is more noticeable if the target strain’s growth performance is already low):

6 $$y=\frac{{K}_{c}}{{K}_{a}},$$

Additional possible representatives for growth efficiency, and other biotic factors that can drive the interaction described above, are discussed in S. VI.

### Adaptive traits

Byproduct synthesis and excretion can be physiologically cheap^22^, and affect negligibly the survival probability of the producer strain, whereas intraspecific competition, always present, shapes the long-term behavior of the phenotypes. The latter is, therefore, the main source of evolutionary pressure on each phenotype in our system. Because the maximum growth rate and the half-saturation constant drive competition, we assume that each phenotype *i* is characterized by ${\mu }_{ma{x}_{i}}$ and *K*_*i*_ (only adaptive traits), with its primary resource indicating which strain the phenotype belongs to. Both traits are linked by a trade-off, ${\mu }_{{\max }_{i}}={\mu }_{ref}\frac{\mathrm{ln}({K}_{i}/{K}_{ref})}{\mathrm{ln}({K}_{i}/{K}_{ref})+1}$, where *μ*_*ref*_ and *K*_*ref*_ are constants^23^. The yield factor, *Y*, does not play any role in the competitive ability of the organism and fitness; for simplicity, we assume that *Y* is identical for all strains. See S.I for more details.

### Eco-evolutionary simulations

The ecological dynamics in our 3-strain system consist of the intraspecific interactions (Eqs (1)–(2)), coupled across strains through a non-transitive cycle via *R* (Eq. (4)) and the chosen driver, *y*. The evolutionary dynamics result from random mutations within each strain, which generate new phenotypes (new populations within the strain that differ in the value of the adaptive trait)^24,25^. Each phenotype’s mutation probability depends on a mutation rate as well as the number of individuals per generation.

Starting from one single phenotype population per strain, mutant phenotypes enter the system at random times during the ecological dynamics. Intraspecific competition drives some phenotypes to extinction whereas other mutant phenotypes are created, triggering a succession of dominant phenotypes that approaches an evolutionarily stationary state (ESS) for each strain^6,26^. Focusing on, e.g. strain *a*, such ESS (Eqs (S4)–(S5)) is an evolutionary target that will remain constant for as long as the performance of *c*, which controls *y*_*c*_ and therefore *R*_*a*_, does not change significantly. Therefore, trait evolution affects the interaction among strains which, in turn, affects cell growth, defining an eco-evolutionary feedback loop.

To ensure equivalent initial conditions for all drivers we studied, we used an initializing period of ∼1000 days in which we impose *R* = 1 to lead all iterations of the system to a same initial stationary state; after that period, *R* becomes dynamic (Eq. (4)). This initialization method does not influence the outcome of the simulation.

### Evolutionary lag and biodiversity measures

The producer strain is a key part of the target strain’s environment. Thus, evolutionary changes for the producer strain result in changes in the evolutionary target (or potential ESS) for the target strain. To quantify how challenging the new environment is for the target strain’s phenotypes (and, therefore, the strength of the selection pressure), we use the evolutionary lag^4^, or relative difference between the strain’s trait value and the ESS. For the maximum growth rate, ${L}_{{\mu }_{max}}(t)=1-\frac{{\mu }_{max}(t)}{{\mu }_{ma{x}_{ESS}}(R(t))}$, (with the denominator provided by Eq. (S5). The emergence of RQ dynamics can affect also the strain number^4^, (which changes with time), *S*(*t*). We monitor these observables as well as the genetic diversity present in the ecosystem. Because we assume that phenotypic differences have only a genetic origin, we refer indistinctly to genotype and phenotype. We use three different biodiversity measures, namely the Shannon index (*I*_*Sh*_, Eq. (S16)), and the standard deviation (*I*_*stdev*_) and inter-quartile range (*I*_*IQR*_) of the adaptive trait distribution (the latter defined as the weighted probability distribution of trait values present within each strain at each time).

## Results

**Figure 1:**
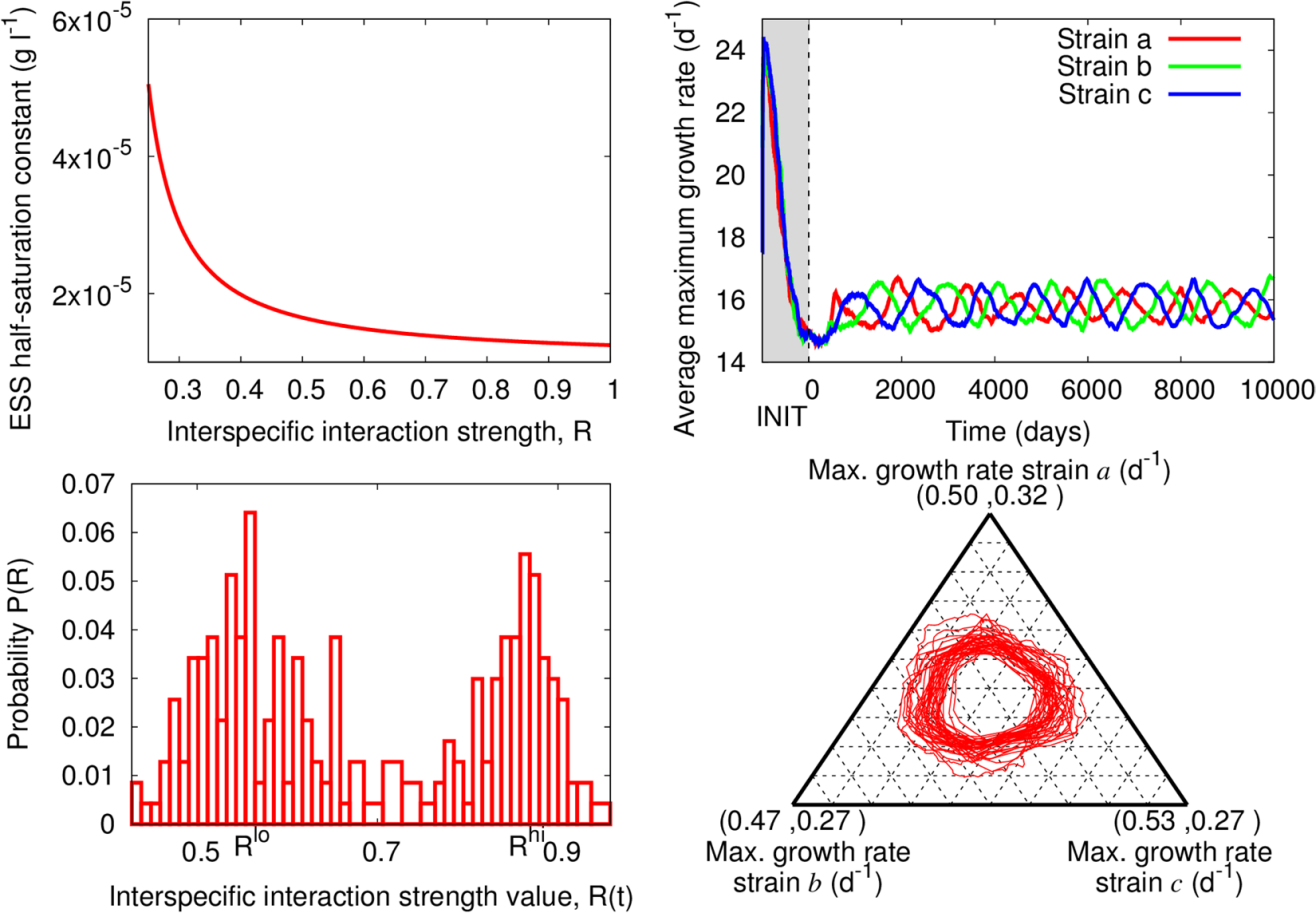
*K*-dependent biotic driver, Eq. (5). Upper Left: Dependence of the half-saturation constant that minimizes resource requirement (i.e. the ESS, Eq. (S5)) on the interaction strength, *R*. Upper Right: RQ dynamics emerging from the 3-strain dynamics when *k* = 5 and *y*_*ref*_ = 2.5, shown for the *μ*_*max*_ of the average phenotype within the strain (shaded area: initialization period). Lower Left: Probability distribution for *R*, showing bimodality with peaks around *R* ^*lo*^ and *R* ^*hi*^. Lower Right: Ternary plot showing the normalized value of the average adaptive trait for the three strains, after removing the initial transient.

In the theoretical system proposed here, each strain’s evolutionary target (or potential ESS) is given by the phenotype with the minimum resource requirement; resource requirement is inversely correlated with fitness and, therefore, it is a measure of competitive ability^26^ (Fig. S3, left). This target, Eq. (S5), is influenced by the effect of the interspecific interaction, encoded in *R*. Larger *R* values select for phenotypes that require a smaller amount of nutrient. These phenotypes show also an enhanced growth efficiency, i.e. smaller *K* (Fig. 1 upper left panel, and Eq. (S1)), which allows cells to reach their maximum growth potential for smaller nutrient concentrations. These results are independent from the specific functional form for *R* (see analytical calculations in S. II). Due to the dynamic nature of the interspecific interaction, however, this target may or may not be realized. The latter case leads here to the RQ.

For drivers like Eq.(5), and with the help of the recursive method explained in S. V, we explored the areas of the (*k*_*R*_, *y*_*ref*_) space for which an ESS is reached and those in which RQ dynamics emerge (Fig. S3, right, Fig. S4, left, and Fig. 1, upper right). Different normalization factors for the driver altered the location of the RQ zones of the parameter space. These trait oscillations are shown in Fig. 1 for the maximum growth rate, *μ*_*max*_, using the parametrization from Table 1. We show this adaptive trait as representative of the evolutionary dynamics because, due to the existing trade-off (Eq. (S1)), the results for *K* are qualitatively identical. The emerging RQ dynamics were identified as oscillations in the intraspecific average value for the adaptive trait. The oscillations for the 3 strains were correlated, connected through the interspecific interaction via the *R* function (in Fig. 1, driven by growth efficiency), which repeatedly visited *R* ^*lo*^ and *R* ^*hi*^, effective minimum and maximum values for the interaction strength (Fig. 1, lower-left panel). These recurring changes in the average trait value matched alternations in dominance occurring within each strain suggesting that, at each time, the corresponding dominant phenotype was overwhelmingly represented in the population (Fig. S4, right). Because strains explore the phenotypic space via mutations, the resulting RQ oscillations were irregular (Fig. 1, lower-right panel). In addition, different drivers generated different *R* ^*lo*^ and *R* ^*hi*^, values that determined the amplitude of the oscillations (i.e. ranges of possible ESS values, see Fig. 1, upper left). These RQ dynamics were not accompanied by population-density oscillations or synchrony (Fig. S5). Population densities fluctuated stochastically around a stationary value in which an increase in density for one strain was not necessarily linked to a decrease in the density of any of the remaining strains. On the other hand, biotic drivers that depend on any of the rapidly-changing variables such as nutrient availability or population number (Eqs (S13)–(S15)) led to a rapidly-changing *R* function that generated oscillations for the ecological variables (e.g. *N*_*i*_) but not for the adaptive traits. For the latter drivers, the traits reached stasis. RQ oscillations could be recovered in these cases, however, when longer periods (e.g. several days) were imposed for changes in such biotic driver (not shown).

**Figure 2:**
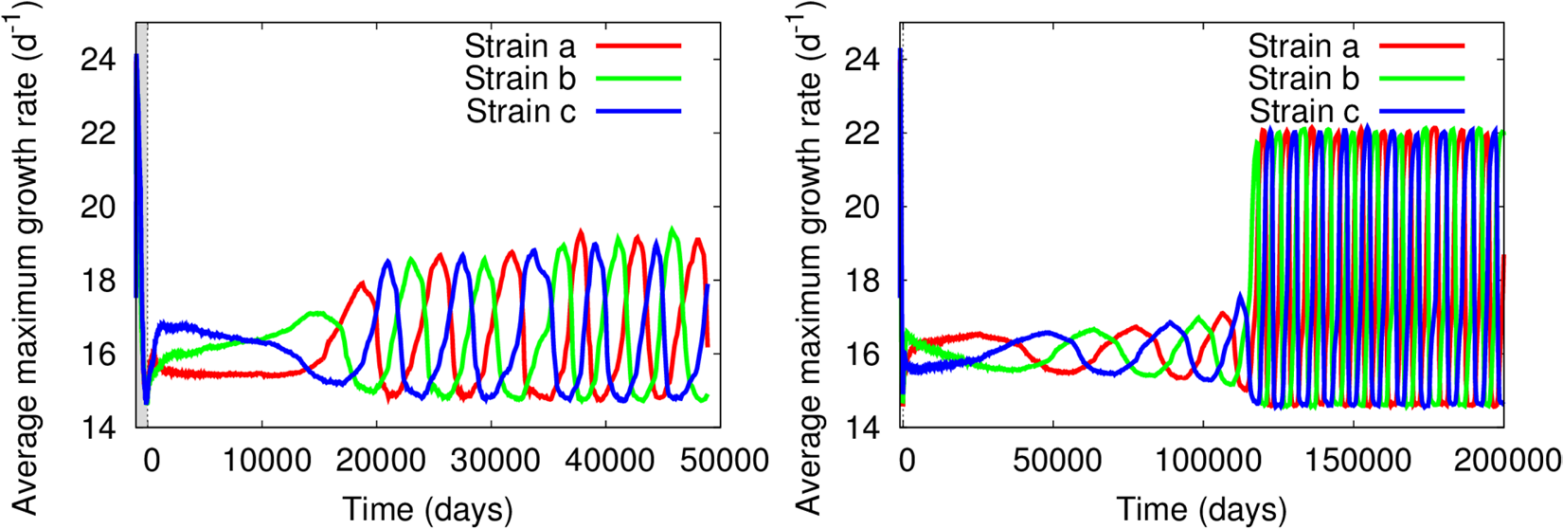
RQ dynamics driven by pairwise drivers with *k*_*R*_ = 10 and *y*_*ref*_ = 1. Left: Biotic driver *y* given by the ratio of maximum growth rates, Eq. (S9). Right: Driver provided by the relative affinity, Eq. (S10), showing a 10^5^ day transient; other replicates show shorter or longer transients to reach the same stationary RQ oscillations.

For all pairwise biotic drivers (Eqs (6), (S9) and (S10)), fixing generically *y*_*ref*_ = 1 (i.e. equal performance for both strains) and the shape/asymmetry parameter to the intermediate value *k*_*R*_ = 10 led to RQ dynamics (Fig. 2); low values for *k*_*R*_ led to stasis. Oscillations triggered by these drivers showed a very heterogeneous timing. Different replicates of the same system showed hugely-different transients, sometimes as short as the initialization period and other cases requiring thousands of days to reach the stationary oscillations. In all cases, amplitudes during the initial transient were small and the associated period long, difficult to discern from stasis. The period of the oscillations progressively shortened while the amplitude increased until, eventually, stationary oscillations were reached. For some drivers, *R* ^*lo*^ and *R* ^*hi*^ and associated ranges for the adaptive traits were given by effective values different from the extreme ones (Fig. 2, left). For other pairwise drivers, RQ oscillations ranged any possible value for the trait allowed by the feasibility conditions, i.e. *R* ^*lo*^ = *R*_*min*_ and *R* ^*hi*^ = *R*_*max*_ (Fig. 2, right).

Both amplitude and period of the RQ oscillations showed a close link with the evolutionary lag, *L*(*t*), and the number of phenotypes per strain, *S*(*t*). For oscillations with long periods and small amplitude (e.g. long transients in Fig. 2), and for stasis, S grew monotonically with no apparent saturation (for the length of our simulations) and *L*(*t*) stayed at a constant, low value (Fig. S6, left). This behavior was also observed during the initialization transient in all cases (Fig. S6 for *t* < 1000). When the RQ regime was reached, however, the number of phenotypes showed irregular oscillations formed by *S*(*t*) increases followed by sharp declines, with the lag function showing spikes that matched dips in *S*(*t*) and vice versa. For sufficiently-high lags, the decline in *S*(*t*) led to the strain’s extinction, breaking in this way the non-transitive cycle (Fig. S6, right panel). This collapse points to a mismatch between the strain’s adaptation rate and the rate of change of its environment. We confirmed this hypothesis by running “rescue” simulations in which we introduced a phenotype matching the ESS when strains experienced large values of the lag. This evolutionary rescue instantaneously reduced lags to zero, thus allowing strains to stay in the system.

**Figure 3:**
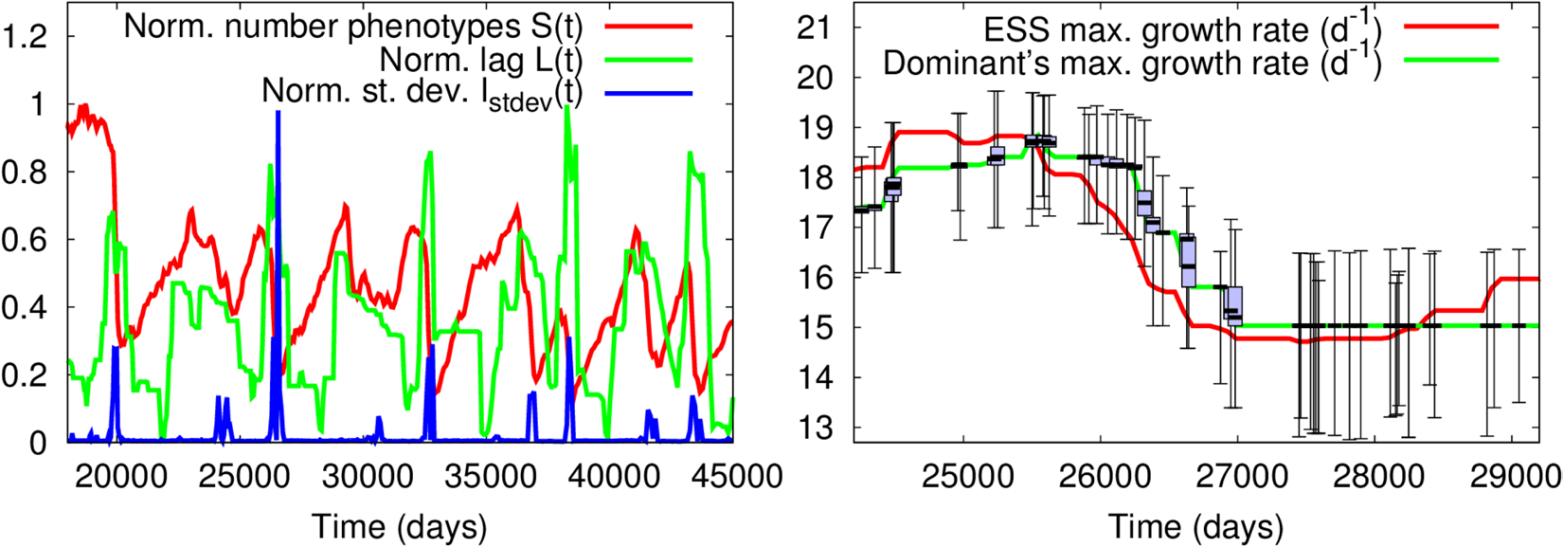
Detail of typical dynamics for a strain at the RQ regime. Left: Number of phenotypes per species, and lag and biodiversity for *μ*_*max*_; to facilitate comparison, the three observables are normalized by their maximum value in that window. Right: Detail of the dynamics on the left panel, showing changes in genetic variability and distribution (box size) as a function of time.

The phenomenology above suggests a close relationship between RQ oscillations and genetic diversity, I(t) (equivalently, phenotypic diversity, see Methods). Indeed, Fig. 3 (left panel) shows a coincidence between large variations in *S*, *L*, and *I*_*stdev*_. Unexpectedly, a decrease in the number of phenotypes within the strain led to a peak in genetic diversity. To clarify this point, we monitored the changes in location and spread of the intraspecific genetic distribution. Figure 3 (right panel) shows boxplots for a representative time window taken from the left panel. Each box’s height indicates the distance between first and third quartile for such distribution, with the median represented by the thick line within the box (whiskers represent the maximum and the minimum within the distribution). Periods with low lag showed very narrow distributions (thin boxes) heavily centered around a clear dominant phenotype; in turn, peaks in lag were associated with wide distributions with no clear dominant phenotype.

Different forms for the biotic driver and interspecific interaction strength function showed similar results (see S. VI and S. VIII).

## Discussion

In stable environments, one single winner is expected when many different organisms compete for one single resource^26^. Moreover, there exists an optimal strategy able to outcompete any other phenotype, the ESS^27^ (S. II). Here, we have used these well-known results to devise a generic system in which eco-evolutionary interactions generate RQ oscillations.

**Figure 4:**
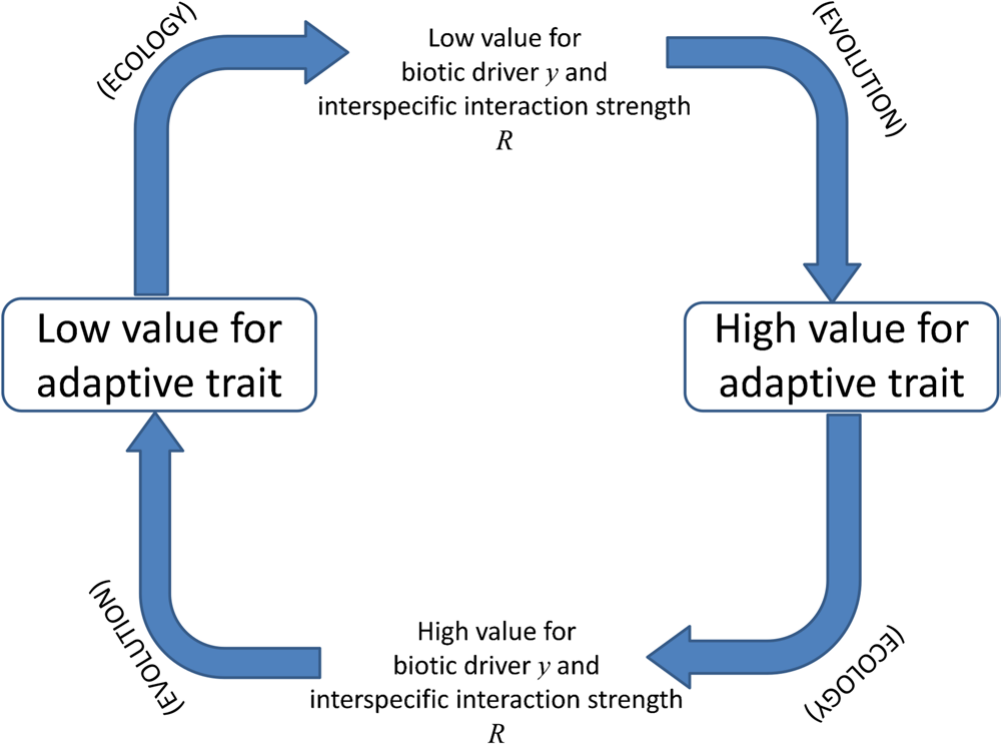
Summary of the eco-evolutionary feedback loop generating RQ dynamics.

Evolution is manifested here as an intraspecific alternation in dominance through mutation and competition. Evolution affects a focal strain’s growth efficiency and ratio of synthesized metabolic byproducts, thus altering the inhibition of a target strain’s growth. A chain of such interspecific interactions forms an ecological non-transitive cycle through which, after the completion of the cycle, the focal strain’s adaptation reverts its evolutionary gradient, closing in this way an eco-evolutionary feedback loop ultimately responsible for the RQ dynamics (Fig. 4). Therefore, these oscillations emerge from an indirect interaction between strains, as the evolution of the focal strain alters the environment of the target strain, which in turn triggers the target strain’s adaptive response.

In our system, evolutionary oscillations are not accompanied by population density ones because the metabolite ratio availability, and not the metabolites per se, generates inhibition and correlates with the producer’s growth performance/efficiency; there is an evolutionary driver for the ecological (non-transitive) interspecific interactions. Note that, although necessary to sustain them, the ecological non-transitive cycle does not suffice to generate these RQ oscillations (drivers such as, e.g. Eqs (S11–S15) do not show RQ dynamics although they show population density ones, as discussed below). The RQ emerges because the ecological non-transitive cycle affects the target strain’s environment (and selection pressure) only after the producer strain has changed significantly and, with it, the biotic driver. In addition, upon completion of a cycle, evolutionary and ecological pressures push a strain in opposite directions, eventually giving rise to RQ oscillations (Fig. 4). On one hand, intraspecific evolution leads to increasing competitive ability within the strain by reducing resource requirement and half-saturation constant (and maximum growth rate, which sets a gleaner versus opportunist trade-off^28^). On the other hand, these smaller and smaller trait values translate into smaller and smaller *R* (higher inhibition), which in turn select for larger and larger values for those very same traits. RQ oscillations emerge from strains trying to reach those two changing evolutionary targets (imposed by *R* ^*hi*^ and *R* ^*lo*^, Fig. 1 lower left panel). As an example, when strain *a* is subject to *R*_*a*_ = *R* ^*hi*^, evolution will lead its adaptive traits towards low values, thus reducing *R*_*b*_ to *R* ^*lo*^. In consequence, for strain *b* high values of the traits will be selected for, which will lead to *R*_*c*_ = *R* ^*hi*^ and, therefore, to low values for strain *c*’s adaptive traits, resulting in *R*_*a*_ = *R* ^*lo*^. Thus, after the completion of the cycle, the evolutionary gradient for *a* is reverted (section S. III and Fig. S2).

Importantly, some time delay needs to be associated with this change of sign of the evolutionary gradient, as the strain requires time to explore the phenotypic space and generate sufficient genetic diversity to track significant changes in the evolutionary target. Because our drivers depend on the strain’s representative value for the adaptive traits, significant changes in the driver only occur when there is an alternation in dominance. Note that, because the dominant is also the most abundant phenotype within the strain, experiments sampling the population to measure each strain’s trait value should be able to observe these RQ dynamics.

A lack of a delay is precisely the reason why we cannot observe RQ dynamics in biotic drivers that are too sensitive to changes in the environment (Eqs (S12)–(S15)). In these cases, quick changes in *R* happen in ecological time and therefore can be interpreted as acclimation responses that obviate the need for the population to adapt evolutionarily. The system tends towards an ESS that averages over time these fast changes in the fitness landscape.

Our results highlight the role of the RQ as key regulator of biodiversity across evolutionary scales. Differently from past work^10^, biodiversity does not refer here to the number of coexisting strains, fixed in our system from the outset, but to the spread of the intraspecific trait distribution. Between changes in the evolutionary target, the phenotypes that are far from the ESS quickly go extinct as the constant environment selects for the fittest phenotypes. The dominant phenotype is found in such large numbers that most mutations result from this or close phenotypes, which can coexist for a considerably long time. Thus, the genetic distribution is heavily centered around the dominant genotype/phenotype while remaining long-tailed due to unusual mutants (thin boxes with long whiskers in Fig. 3, right panel). A change in the environment (i.e. change in *R*) sets a new evolutionary target, normally challenging enough (i.e. different enough from the previous ESS) to dethrone the ruling dominant and close phenotypes. As a measure of selection pressure, high lags indicate highly-challenging conditions for each phenotype within the strain. The larger the lag at the moment of change, the more dramatic the selection process. Thus, lag peaks match changes in ESS that trigger dominance alternation shortly after. RQ dynamics and associated changes in evolutionary target provide new phenotypes with the chance to thrive and replicate, challenging dominance. Without a clear dominant phenotype, the spread of the genetic distribution increases in transitional stages, reason why we observe the “paradoxical” decrease in the number of phenotypes per strain (*S*(*t*)) matching peaks in lag (*L*(*t*)) and biodiversity (*I*(*t*)). Conversely, *S* rises monotonically during periods in which the environment remains constant, but biodiversity does not increase because most of the new mutants will be phenotypically-close to the dominant. We propose that both *S* and *L* can be used as indicators of the emergence of RQ dynamics, which opens new and exciting possibilities to characterize the RQ in experiments.

In addition, RQ dynamics contribute to the resilience of the ecosystem. Environmental changes renew the adaptive landscape and constitute an opportunity for the creation of biodiversity. Biodiversity acts as a buffer that may allow the strain to respond to the changes in the evolutionary target imposed by the interspecific interaction. In consequence, long periods around the same ESS make the strain less resilient against environmental change due to the reduced genetic diversity.

Note that our RQ oscillations do not result from negative frequency dependence^29^, as it is not “being rare” that matters but being different from the current dominant in the “right way”. In spite of the peaks in biodiversity after a dominant goes extinct, the difference between previous and new environmental conditions can be too sharp (sudden large lag peaks) for the strain to “find” the right phenotype in time to avoid extinction. On the other hand, low lags accompany oscillations with low amplitude and period because the evolutionary process is generating enough genetic diversity for the strain to catch up quickly with such changes (e.g. low amplitudes observed in the transients of Fig. 2).

The irregular character of the amplitude and timing of our RQ oscillations stems from natural selection acting on phenotypes obtained from random mutations. For the biotic drivers Eq. (5) and (S7)–(S8), the amplitude is mostly determined by the parametrization of the *R* function, which determines the realized extremes *R* ^*lo*^, and *R* ^*hi*^. The variability in the transient observed for some pairwise biotic drivers (Eqs (6) and (S9)–(S10)) reflects the irregular, random way in which incipient RQ oscillations progressively increase in amplitude to reach the realized trait extremes (motivated by *R* ^*lo*^ and *R* ^*hi*^). Such increase could result from a stochastic amplification of the oscillations, but without any analytical basis the underlying mechanism remains unclear.

We have focused, for the sake of concreteness, on one specific experimental realization. However, the basic principle underlying the RQ oscillations for our 3 bacterial strains is general. Our model suggests that natural or synthetic systems can show evolutionary oscillations even if species do not interact directly; it would suffice if one species’ evolution alters negatively the environment of only the next species from a closed (odd) chain. Moreover, intraspecific competition drives each strain’s evolution in our system, but any fitness-related changes/mechanisms may alternatively trigger the evolutionary cycle we identify here as the RQ. On the other hand, ecological non-transitive cycles like the one described here can be engineered in multiple ways in many microbial systems (see S. III). Because our RQ-inducing drivers depend on traits, lab strains should not lose the engineered mechanisms and, therefore, they need to be coupled to essential cell components. More generally, we propose that sufficiently-asymmetric drivers related to pairwise interactions as the ones described above generically trigger emergent RQ oscillations that self-organize to their maximum amplitude with no additional fine-tuning. In experiments, the *R* function and associated parameters will be heavily constrained by the type of biotic driver and interaction set up during the genetic engineering that produces the experimental layout. Different shapes and parameters for the interaction function *R* do not alter qualitatively the results above. Other parameters are fixed by choices such as the duration or strength of the forcing period (which do not alter any of the results above, qualitatively or quantitatively), the dilution rate or resource input concentration (which constrain the feasibility conditions and the ranges for the trait amplitude in the RQ regime), or type of resource (which determines the yield factor, *Y*, and the trade-off reference values and form, just changing the target ESS for each strain and ranges for the RQ oscillations). We could have also considered drivers that are positively correlated with growth efficiency (e.g. focusing on growth-enhancing aminoacids excreted for optimal growth performance, S. III), in which case the non-transitive cycle would have required a negative correlation between the *R* function and the driver.

Ultimately, our theoretical framework can be used to predict whether a particular experimental setup will be able to show RQ dynamics, to inform such experiment and help in its design, and extend it to, e.g. longer time scales that cannot be achieved otherwise. Our model can also go beyond experiments studying aspects such as the strength of the evolutionary pressure (i.e. lag) and biodiversity. Our framework can indeed help discern whether associated experimental observables correspond to real versus apparent stasis; for example, RQ dynamics where the amplitude is small and the period long. We hope our work will inspire and inform experiments with which unravel the mysteries of the RQ.

## Electronic supplementary material

Supplementary Information
