# Supplementary material for: Eco-evolutionary Red Queen dynamics regulate biodiversity in a metabolite-driven microbial system
Source: Sci Rep. 2017 Dec 15;7:17655. doi: 10.1038/s41598-017-17774-4 (PMC5732168; doi:10.1038/s41598-017-17774-4)
Supplement: Supplementary file 1 — Supplementary Information [file 41598_2017_17774_MOESM1_ESM.pdf]

Eco-evolutionary Red Queen dynamics regulate biodiversity in a metabolite-driven microbial system

Supplementary Information

Juan A. Bonachela*†[1], Meike T. Wortel[2], and Nils Chr. Stenseth[2]

[1]Marine Population Modeling Group, Department of Mathematics and Statistics, University of Strathclyde, Glasgow G1 1XH, Scotland,UK.
[2]Centre for Ecological and Evolutionary Synthesis (CEES), Department of Biosciences, University of Oslo, PO Box 1066 Blindern, Oslo 0316, Norway

S.I Trade-off function

The maximum growth rate, μ_{max_a}, and the half-saturation constant, K_a, are linked by a trade-off that depends on the type of microbe and resource it feeds upon. In our model, strains feed on different resources; however, due to the lack of information about the trade-off associated with different sugars (and to highlight the generality of our results), we use for all three strains the functional form for *E. coli* growing on glucose [1]:

$$\mu_{max_i} = \mu_{ref} \frac{\ln (K_i/K_{ref})}{\ln (K_i/K_{ref}) + 1} \tag{S1}$$

where μ_{ref} and K_{ref} are reference values that depend on the specific resource and species under study (see Table 1 for values used here). Our results do not depend qualitatively on the shape or parametrization of this trade-off as long as the trade-off holds, i.e. there is a positive link between the two traits. See S.VIII.

S.II Ecological and evolutionary steady state for intraspecific competition

Without loss of generality, let us focus on strain a hereon. In the model defined by equations Eq.(2)-(3), the chemostat conditions ensure reaching a stationary state, given by:

*To whom correspondence should be addressed: juan.bonachela@rutgers.edu
†Current address: Department of Ecology, Evolution, and Natural Resources, 14 College Farm Rd, New Brunswick, NJ 08901, USA.

$$[A]_{st} = \frac{w\,K_a}{R_a \mu_{max_a} - w} \tag{S2}$$

for the nutrient (note the feasibility condition $R_a \mu_{max_a} > w$), and:

$$N_{st} = Y_a \left([A]_{input} - [A]_{st}\right) \tag{S3}$$

for the population of cells.

Thus, if K (conversely, μ_{max}) is the only adaptive trait (see main text), and with competition as only possible interaction among different phenotypes of the focal strain a, the only possible result is one single dominant phenotype: the one that requires the least amount of resources [2], as competitive ability is inversely correlated with resource requirement. We can calculate this *evolutionarily stable strategy* (ESS) [3], by calculating the value of the adaptive trait that minimizes resources at the stationary state, that is:

$$\frac{d[A]_{st}}{dK_a} = 0;$$

Using Eq.(S1) and defining $z = \log\left(K_a/K_{ref}\right)$, the condition given by Eq.(S4) can be expressed as an equation for z whose solution is given by:

$$z^* = \frac{(2w - R_a\mu_{ref}) + \sqrt{(R_a\mu_{ref} - 2w)^2 + 4\left(R_a^2\mu_{ref}^2 - w^2\right)}}{2\left(R_a\mu_{ref} - w\right)} \tag{S4}$$

(note the feasibility condition $R_a > 4w/(5\mu_{ref})$) and the ESS therefore can be written as:

$$\begin{aligned} \mu_{max_{a_{ESS}}} &= \mu_{ref}\frac{z^*}{z^*+1} \\ K_{a_{ESS}} &= K_{ref}e^{z^*}. \end{aligned} \tag{S5}$$

The resource requirement is thus given by:

$$[A]_{st_{ESS}} = \frac{w\,K_{a_{ESS}}}{\mu_{max_{a_{ESS}}} - w} \tag{S6}$$

which depends on the adaptive traits and the dilution rate. Note that, following this equation the yield factor, Y, does not play any role in the competitive ability of the organism; z^* is mainly determined by the trade-off reference values, and by the environment (through the interspecific interaction strength and the dilution rate, biotic and abiotic factors respectively). Changes in R_a (in this case, triggered by strain c) will thus alter strain a's evolutionary target.

For a fixed R, the ESS above is also convergence-stable. Therefore, with one single strain the only possible long-term evolutionary outcome possible under chemostat conditions is an ESS, which can be interpreted as stasis and, therefore, opposite to Red Queen (RQ) dynamics.

S.III Non-transitive cycle

Our system prevents the collapse or divergence of the co-existing strains through an interspecific non-transitive cycle that is regulated/modified by the outcome of intraspecific competition for strain-specific resources. Such cycle can be engineered in many ways in the laboratory.

In the example discussed in the main text, good growth performance for, e.g. a (i.e. low y_a) leads to a high inhibitor ratio, which translates into a low $R_b(y_a)$ and the consequent reduction in strain b's growth. Such inhibition, in turn, gives rise to an evolutionary pressure selecting for lower growth performance for phenotypes belonging to strain b (high y_b) and, thus, low inhibitor ratio leading to a high $R_c(y_b)$) representing the lack of effect on strain c's growth. Thus, phenotypes from strain c can grow at a high rate, which facilitates high inhibitor ratios that will reduce strain a's growth, closing in this way the non-transitive cycle (see Fig.S2). After the completion of a period, the cycle can be summarized as a loop from the originating strain to itself, which can be seen in the figure by omitting the shaded area.

In addition to the examples outlined in the main text, there are many other possibilities to engineer cells to obtain such trait-based non-transitive cycle. For example, for *Bacillus subtilis* glutamate utilization requires the presence of arginine [4], another metabolic byproduct; or the cycle may be based on the use of byproducts that are synthesized under growth-stress conditions (e.g. glutamate, an alternative source of nutrients excreted under growth-stress conditions, i.e. poor growth performance [5]). Another possibility would be the interspecific activation of the transporter system: A activates b's transport system and therefore facilitates/enhances the uptake of B (e.g. arabinose-activated synthesis of transporters that take up also galactose [6]). Or even the possibility to take up byproducts from other strains that prevent futile cycles and therefore improve growth in the "target" strain.

S.IV Parametrization

The extremes of the interaction function reveal how dramatic the effect of the producer strain, e.g. c, is upon the target strain's growth. Choosing $R_{top} = 0.75$ and $R_{min} = 1 - R_{top}$, for example, phenotype a can only reach maximal levels, μ_{max}, if $R_a(y_c) = 1$, but maintains a minimal growth even if the biotic driver is not strong enough to trigger interaction.

The choice of focal organism and physical environment (see Table 1) leaves only k_R and y_{ref} as free parameters. The latter indicates when the biotic driver triggers non-trivial interaction strengths (i.e. y such that $R \ncong R_{min}$ and $R \ncong R_{max} = R_{top} + R_{min}$), while k_R represents the susceptibility of the target strain or, in the case of a pairwise biotic driver (e.g. Eq.(6)), the degree of asymmetry between the two interacting strains that influences the dynamics of the target strain.

Following the exploratory method described below, we can find the (y_{ref},k_R) that give rise to RQ dynamics, at least for cases in which the driver depends only on the producer strain.

S.V Exploratory method to find the RQ

In some cases, it is possible to explore the parameter space by mapping the non-transitive cycle into a recursion for each strain, which facilitates a tentative prediction of the pairs (k_R, y_{ref}) for which RQ dynamics emerge. When the biotic driver depends only on the producer strain (e.g. Eq.(5)), the (negative) influence of a over b is qualitatively similar to the (negative) influence of c over a, and the whole non-transitive cycle can be qualitatively summarized as the effects of a over itself (see Fig.S2), $R_a(y_a(t))$. Thus, given an initial value for R_a, we calculate the associated ESS using Eqs.(S5); then, we calculate accordingly the new y_a using, e.g. Eq.(5), which in turn will determine a new value for R_a following Eq.(4), and so on. If such iteration leads to a stationary value, the pair (k_R, y_{ref}) potentially provides stasis for the complete 3-strain system; otherwise, sustained oscillations indicate potential RQ dynamics (see, e.g. Fig.S3 right).

On the other hand, the multi-strain dependence of y for pairwise drivers prevents a meaningful definition of the recursion $R_a(y_a)$, and therefore the exploratory method cannot be used to find the appropriate (k_R, y_{ref}) that enable RQ dynamics. We observed, however, RQ dynamics for all such pairwise drivers after generically setting $y_{ref} = 1$ and $k_R = 10$ in these cases.

S.VI Explicit functional forms for the driver

In the main text, we assume a positive relationship between the interaction strength R and its driver y (see Fig.S1), which in turn is negatively correlated with growth performance (see previous section). With this in mind, let us now enumerate a suite of possible forms for the driver that could represent such an interaction. Given the simplicity of our system, any interaction needs to include necessarily either the main traits and/or resources, which means that they will form part somehow of the biotic driver for the interaction strength.
i) Strain-specific performance driven factor: In this group we can find cases in which the biotic factor that drives the interspecific interaction depends singly on the producer strain's performance. Because a bad performance translates into a smaller ratio of the inhibiting byproducts, the first option for the functional form for y is provided by Eq.(5), showing a positive correlation with K. We can also consider other definitions for "bad performance", for example defined as the need to increase the uptake rate potential to maintain the same growth rate; in this case, if a is the target strain and c the producer strain, y would be:

$$y_c = \frac{\mu_{max_c}}{\mu_{max_{c_{ESS}}}(R=1)}. \tag{S7}$$

where the normalization factor is, in this case, the maximum uptake rate in the best possible scenario for the producer strain ($R = 1$). Another valid normalization factor could be the reference value used for the trade-off, μ_{ref}. This choice does not affect qualitatively our results.

An additional definition for bad performance considers low affinity for the primary resource. Affinity measures the efficiency of the uptake process. Therefore, a poor affinity of c for C translates into poor growth performance and therefore increased inhibitor ratio hindering a's growth. Thus, there should be a negative correlation between affinity and

the driver, y. Defining affinity as the ratio of maximum uptake rate and half-saturation constant [7]:

$$y_c = \frac{K_c}{\mu_{max_c}} \left(\frac{K_{c_{ESS}}(R=1)}{\mu_{max_{c_{ESS}}}(R=1)} \right)^{-1}, \tag{S8}$$

where we used the link between uptake and growth to express y_c in terms of growth traits. Because we use as a normalization factor the ESS value for the affinity for $R = 1$, the yield parameter does not appear in this expression.
ii) Relative-performance driven factor: A plausible driver of the interspecific interaction could be relative growth performance, as opposed to individual performance as introduced above (see main text).

Thus, the expressions for y depend, in this scenario, on the traits of both the producer and target strains (e.g. c and a, respectively). In the "negative interaction" scenario necessary for the non-transitive cycle above, the inhibitory effect of c on a is less dramatic if a's growth performance is good. Therefore, if we represent growth performance with the half-saturation constant, the pairwise interaction driver takes the form provided by Eq.(6). If, on the other hand, we represent growth performance using the potential maximum uptake rate:

$$y_c = \frac{\mu_{max_c}}{\mu_{max_a}}, \tag{S9}$$

Finally, if we use nutrient affinity as a proxy for performance:

$$y_c = \frac{K_c}{\mu_{max_c}} \left(\frac{K_a}{\mu_{max_a}} \right)^{-1}. \tag{S10}$$

For such drivers, there is no need for normalization factor to compare results across cases.
iii) Resource-concentration driven factor: In all the cases above, we focus on growth-inhibiting byproducts. If, instead of ratios, it is individual inhibitor concentration that influences the interaction, other factors such as population density can drive the interaction across strains:

$$y_c = \frac{N_c}{N_{c_{ESS}}(R=1)}, \tag{S11}$$

Other possibilities are related with the limiting nutrient being directly the inhibitor for the target strain (e.g. C limiting a's growth), for example:

$$y_c = \frac{[C]}{[C]_{ESS}(R=1)} \tag{S12}$$

or the classic mathematical representation for nutrients that act as (non-competitive) inhibitors:

$$y_c = \frac{[C]}{K_a}. \tag{S13}$$

This expression gauges how available the inhibiting resource C is with respect to how efficient is a growing on its own nutrient. Finally, in terms of relative resource availability or relative biomass:

$$y_c = \frac{[C]}{[A]} \tag{S14}$$

$$y_c = \frac{N_c}{N_a}. \tag{S15}$$

As explained in the main text, however, the timing of changes in any driver in which concentration (or biomass) participate prevents the emergence of RQ dynamics, due to the short reaction time of the cells allowing the strains to acclimate ecologically without the need to rely on long-term evolutionary adaptation.

S.VII Biodiversity

In addition to monitoring the number of phenotypes per strain, we characterized genetic diversity by keeping track of the phenotype variability within each strain. To this end, we measured the standard deviation and inter-quartile range associated with the intraspecific trait distribution, for which we used the classic definitions [8]. We also measured the Shannon index (used in ecology to measure biodiversity), defined as:

$$I_{Sh_a}(t) = -\sum_{i=1}^{S_a} f_i \log(f_i) \tag{S16}$$

where f_i is the relative frequency of phenotype i within strain a, i.e. $f_i = \frac{N_i}{\sum_i N_i}$. The three indicators of biodiversity, I_{stdev}, I_{IQR}, and I_{Sh}, provided qualitatively-similar results to those shown in Fig.3.

S.VIII Additional results

ESS as a function of R

Fig.S3 shows that the interspecific interaction between strains alters the location of the minimum for resource requirement at the stationary state (a measure of competitive ability [2]); this minimum (which corresponds to the ESS, see S.II), is negatively correlated with R. A negative correlation can be also observed for the ESS value for the adaptive trait as a function of R (Fig.1, upper left). Importantly, these results are independent from the shape for the interaction function, R. Plots like Fig.1 (upper left) can help us determine the range of the changes in the ESS expected for a specific interaction strength interval. The range for the amplitude increases with the dilution rate (although increasing w decreases feasibility, S.II). The smaller the range is, the smaller the amplitude of oscillations and therefore the closer the resulting long-term behavior is to stasis.

Stasis

In the complete model, the stasis regime is highly nontrivial, as it results from the interaction between the three strains, each with a potentially different $R(t)$ and, therefore, each with a potentially different ESS value for their adaptive traits. In Fig.S4 (left panel),

we can see two different examples. In the inset, the three strains all reach the same ESS, which in turn agrees with the best possible scenario within the interval of possible R: the ESS associated with $R = 1$. Note that, in this case, the coupled evolutionary dynamics take the three strains to the (nontrivial) ESS whereas if we fixed R (i.e. constant forcing until the end of the experiment) the three strains would reach such state independently from each other.

In the main panel, we parametrized the trade-off functions with (arbitrary) strain-specific parameters, representing their growth on different nutrients. Due to the lack of information about other sugars' trade-offs, we assumed $\mu_{ref_a} = \mu_{ref}$, $\mu_{ref_b} = 2\mu_{ref}$, and $\mu_{ref_c} = 3\mu_{ref}$. As a result, the strains reach a different ESS. This quantitative differences are the only observed effect of changes in the trade-off function.

Regardless of the final outcome of the dynamics (i.e. RQ or stasis), the strain can be represented by the dominant at any time (see Fig.S4, right panel). Similarly, in the laboratory, sampling each strain will most probably result in obtaining the most abundant (i.e. dominant) phenotype.

One-strain recursion and interaction delay

As mentioned above, the recursion $R_a(y_a)$, i.e. the effect of a over itself, can summarize at least qualitatively the non-transitive cycle. When the same two R^{hi} and R^{lo} are visited by all strains, like in our system, the equivalence is also quantitative. As Fig.S2 points out, in the non-transitive cycle the effect of a over b is qualitatively similar to that of c over a; if each strain visits only the same two values for R (R^{lo} and R^{hi}) every cycle, and those two values agree for all strains, then the effect of a over b is also quantitatively similar to that of c over a, and we can map the cycle exactly into the effect of a over itself.

This recursion is meaningful when the driver depends on only the producer strain. For all the cases in which the biotic driver depends on the performance of one single strain (drivers given by Eqs.(5), and Eq.(S7) and (S8)), the $R_a(y_a(t))$ iteration predicts reliably the emergence of RQ or stasis in the complete model (e.g. Fig.S3, right). However, if the biotic driver depends on any of the rapidly-changing variables such as nutrient availability or population number (Eq.(S13)-Eq.(S15)), the R function changes equally frequently and $R_a(y_a(t))$ fails to predict the evolutionary outcome: oscillations are observed in the yellow regions predicted by the recursion, but only in the ecological variables and not in the adaptive traits, which reach an ESS.

This lack of a sufficient delay is also the reason why a one-strain system cannot show RQ dynamics with our setup, but the one-strain recursion $R_a(y_a)$ is able to predict when the 3-strain system does. Without the complete non-transitive cycle, parametrizations in the yellow areas will give rise to R^{lo} and R^{hi} that are, in one-strain cases, very close to each other (the resulting distribution for R or that of the adaptive trait are, effectively, unimodal as opposed to Fig.1, lower-left panel); the two "environmental conditions" between which the strain switches are therefore very similar, and the two potential dominant phenotypes are present at all times. Thus, alternation, i.e. oscillations occur artificially often and the strain converges to stasis.

Lack of ecological oscillations

As mentioned in the main text, the temporal behavior of the strains' population densities does not show a clear oscillatory trend or synchrony. Indeed, Fig.S5 shows that there is no periodicity in the changes for either the total population density or the dominant's density, whose dynamics are irregular but not oscillatory. Moreover, the increase in one strain's density does not necessarily lead to a decrease in the other two, nor does the number of cells reach almost negligible values like described in other RQ models [9]. The fact that the non-transitive cycle is driven by traits (i.e. evolution), and strains alternate between ESSs, explains why oscillations in the population densities are not needed to maintain the RQ.

Evolutionary rescue

Fig.S6 (left panel) shows that, during the initialization period, the number of phenotypes per strain increases monotonically. However, when the RQ regime is reached, the associated oscillations in the evolutionary lag lead to oscillations in the number of phenotypes, as peaks in lag trigger multiple phenotype extinctions whereas low lag allows for the accumulation of dominant-related phenotypes.

For sufficiently-high evolutionary lag values, the focal strain's biodiversity may not be high enough to "respond" to such a sudden environmental change, which drives the strain to extinction (Fig.S6, right). These cases can be avoided by introducing a new phenotype close to the new evolutionary target thus reducing the lag ("evolutionary rescue"). See main text for further discussion.

Alternative R functions

We tested the dependence of the RQ emergence on the functional form chosen for the interspecific interaction. To this end, we replaced Eq.(4) by the linear function $R = k_R\,(y - y_{ref})$, where there is no upper limit but we establish a lower limit ($R \geq R_{min}$ for any y, with R_{min} fixed such that $R_{min} \geq 0$).

With this linear functional form, we studied the case in which the biotic driver depends on the affinity of the producer strain, Eq.(S8) . The simplified one-strain scenario predicts evolutionary oscillations in a wide region of the parameter space; the 3-strain system shows large lag periods in that region that lead easily to the collapse of the population (results not shown). RQ dynamics emerge, however, when evolutionary rescue is included to perpetuate evolutionary oscillations.

Symbol	Description	Units	Value/Range
N_i	Population density for phenotype i	$cells \cdot L^{-1}$	Ecological variable
μ_{max_i}	Maximum growth rate phenotype i	d^{-1}	Evolutionary variable
K_i	Half-saturation constant phenotype i	$g \cdot L^{-1}$	Evolutionary variable
$[A]$	Concentration for nutrient A	$g \cdot L^{-1}$	Ecological variable
$[A]_{input}$	Input of nutrient A in the chemostat	$g \cdot L^{-1}$	$400 \cdot 10^{-6}$
R_a	Interspecific interaction function for strain a	$-$	Eco-evolutionary variable
y	Biotic driver for the interspecific interaction	Depending on choice	Eco-evolutionary variable
y_{ref}	Modulator for biotic driver in the R function	Same as y	0.5-5
k_R	Steepness for the R function	$-$	1-50
w	Chemostat dilution rate	d^{-1}	4.8
Y	Cell yield factor	$cell \cdot g^{-1}$	$4.96 \cdot 10^{12}$
μ_{ref}	Reference max. growth rate for trade-off function	d^{-1}	32.4
K_{ref}	Reference half-saturation constant for trade-off function	$g \cdot L^{-1}$	$5.5 \cdot 10^{-6}$
R_{top}	Added to R_{min}, max. value interaction function	$-$	0.75
R_{min}	Min. value interaction function	$-$	0.25
p_{mut}	Mutation rate	$mutations \cdot generation^{-1}$	10^{-6}

Table S1: Table of parameters and variables in the system. The first group are phenotype-specific, the second group, strain-specific, the third group depend on the type of biotic driver, and the last group are common to all phenotypes. Typical *E. coli* values for parameters in the last group taken from [1, 10] and our own laboratory reference values.

References

[1] Wirtz, K.W. A generic model for changes in microbial kinetic coefficients, *J. Biotech.* **97**, 147-162 (2002).

[2] Tilman D. *Resource Competition and Community Structure* (Princeton University Press, 1982).

[3] Dercole, F. & Rinaldi, S. *Analysis of Evolutionary Processes: the Adaptive Dynamics Approach and its Applications* (Princeton University Press, 2008).

[4] Commichau, F.M., Gunka, K., Landmann, J.J. & Stülke, J. Glutamate metabolism in *Bacillus subtilis*: gene expression and enzyme activities evolved to avoid futile cycles and to allow rapid responses to perturbations of the system, *J. Bacteriol.* **190**, 3557-3564 (2008).

[5] Burkovski, A., Weil, B. & Krämer, R. Glutamate excretion in *Escherichia coli*: dependency on the relA and spoT genotype, *Arch. Microbiol.* bf 164, 24-28 (1995).

[6] Krispin, O. & Allmansberger, R. The *Bacillus subtilis* AraE protein displays a broad substrate specificity for several different sugars, *J. Bacteriol.* **180**, 3250-3252 (1998).

[7] Healey, F.P. Slope of the Monod Equation as Indicator of Advantage in Nutrient Competition, *Microb. Ecol.* **5**, 281-286 (1980).

[8] Mendenhall, W., Beaver, R.J., & Beaver, B.M. Introduction to probability and statistics. Belmont, CA: Brooks/Cole, Cengage Learning (2009).

[9] Rabajante, J.F., Tubay, J.M., Uehara, T., Morita, S., Ebert, D., & Yoshimura, J. Red Queen dynamics in multi-host and multi-parasite interaction system, *Sci. Rep.* **5**, 10004 (2015).

[10] Lendenmann, U., Snozzi, M., & Egli, T. Kinetics of the simultaneous utilization of sugar mixtures by *Escherichia coli* in continuous culture, *App. Env. Microbiol.* **62**, 1493-1499 (1996).

Supplementary figures

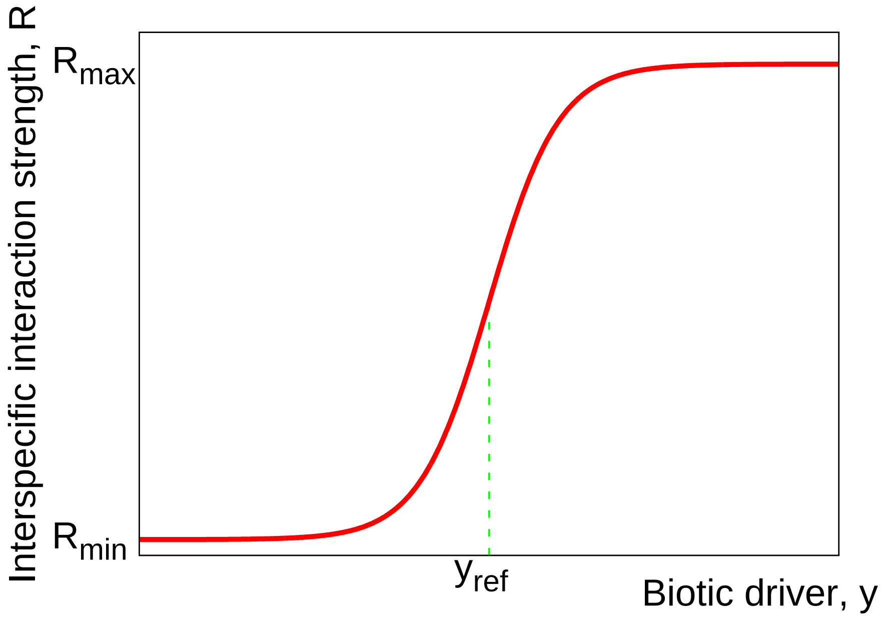

Figure S1: Interspecific interaction strength, R, as a function of the biotic driver value, y, as determined by Eq.(4).

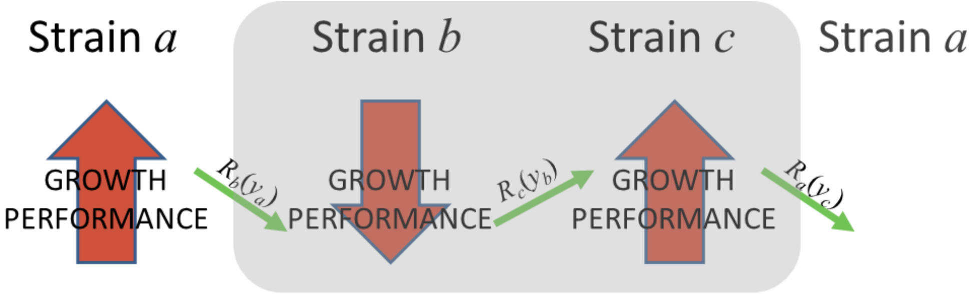

Figure S2: Non-transitive cycle in which evolutionary changes in growth performance for each strain (larger, red arrows) alter the interspecific interaction, change encoded with R (smaller, green arrows).

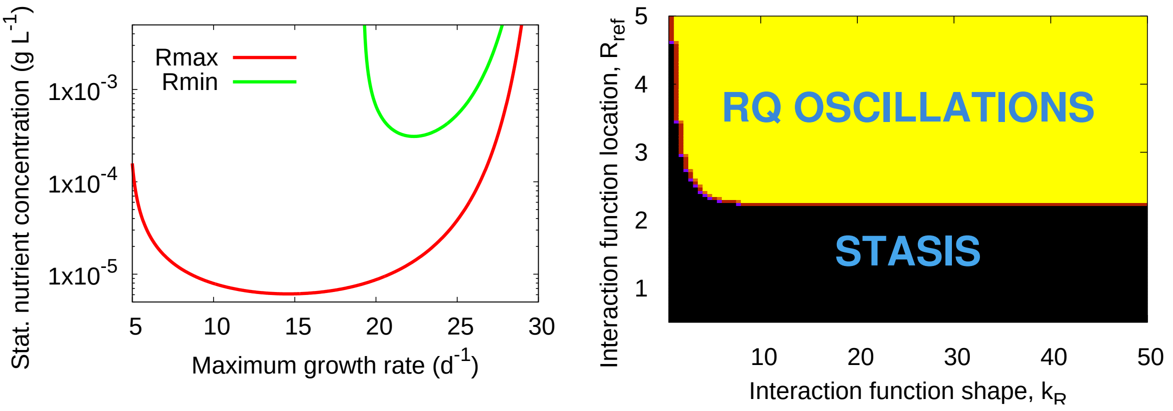

Figure S3: Left: Dependence of the resource requirement at the stationary state (Eq.(S2)) on the maximum growth rate, assuming a constant interspecific interaction strength R fixed at two specific values ($R_{max} = 1$ and $R_{min} = 0.25$). Right: Parameter space showing the potential for RQ dynamics (yellow) and stasis (black) for the K-dependent driver, Eq.(5).

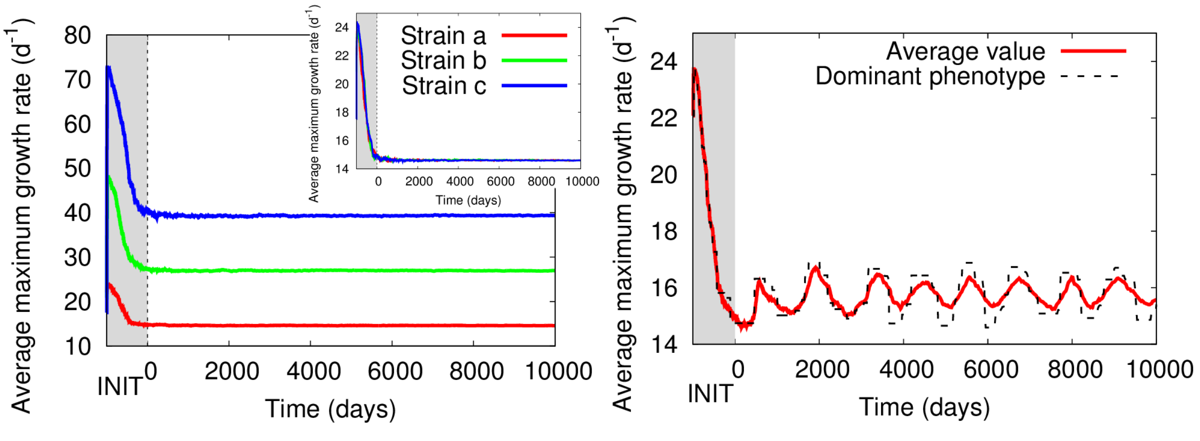

Figure S4: Stasis emerging from the dynamics with three strains when $k = 10$ and $y_{ref} = 1.5$; the three strains interact dynamically until they reach the strain-specific ESS given by Eq.(S5) with μ_{ref} increased by a strain-specific factor (see main text). Inset: Stasis resulting from using the same trade-off parameters for all strains, which results in the same ESS. Right: Strain a evolutionary dynamics for the RQ case in Fig.1, considering the average value for the adaptive trait, μ_{max} (red line) and value for the dominant phenotype (black dashed line).

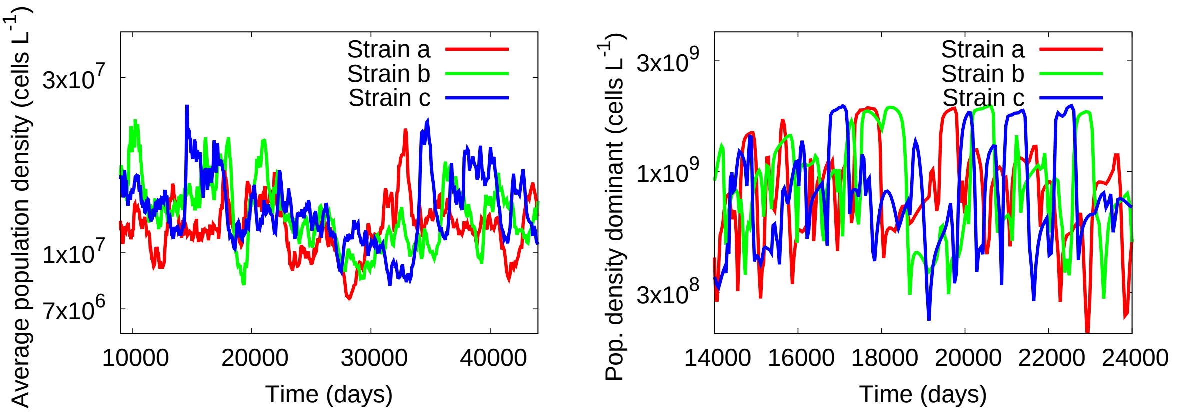

Figure S5: Left: Temporal behavior of the average population density for each strain. Right: Temporal behavior of the population density for each strain's dominant phenotype.

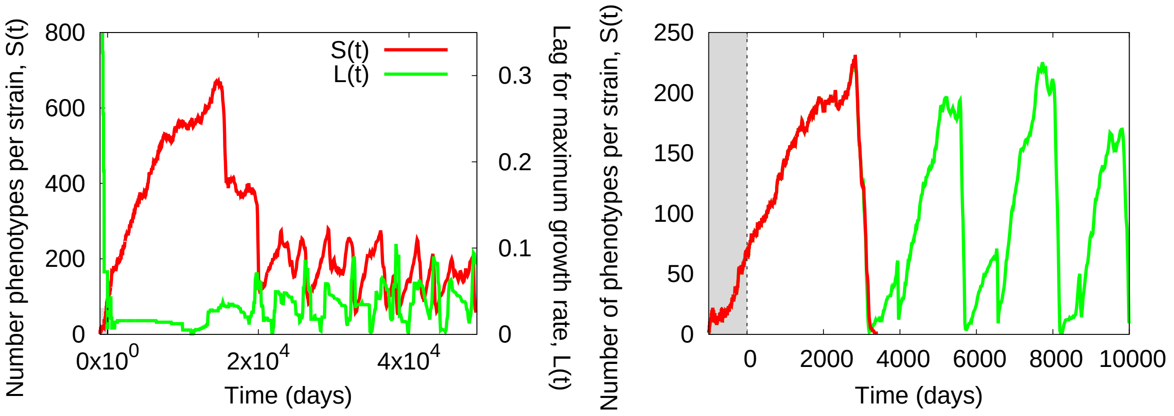

Figure S6: Dynamics for the number of phenotypes within strain a. Left: Number of phenotypes (red line) and associated lag (in this case, for μ_{max}, green line) as a function of time, showing the coincidence of peaks in lag and dips in $S(t)$; the biotic driver is the ratio of maximum growth rates (Eq.(S9)) with $k_R = 10$ and $y_{ref} = 1$. Right: Example of crashing simulation (red line) and "rescued" simulation (green line) for a driver described by Eq.(6) and same R parameters as left panel.
